# Muscleblind-Like 1 Knockout Mice Reveal Novel Splicing Defects in the Myotonic Dystrophy Brain

**DOI:** 10.1371/journal.pone.0033218

**Published:** 2012-03-13

**Authors:** Koichi Suenaga, Kuang-Yung Lee, Masayuki Nakamori, Yoshiki Tatsumi, Masanori P. Takahashi, Harutoshi Fujimura, Kenji Jinnai, Hiroo Yoshikawa, Hongqing Du, Manuel Ares, Maurice S. Swanson, Takashi Kimura

**Affiliations:** 1 Division of Neurology, Department of Internal Medicine, Hyogo College of Medicine, Nishinomiya, Hyogo, Japan; 2 Department of Molecular Genetics and Microbiology, College of Medicine, University of Florida, Gainesville, Florida, United States of America; 3 Department of Neurology, Chang Gung Memorial Hospital, Keelung, Taiwan; 4 Department of Neurology, Osaka University Graduate School of Medicine, Suita, Osaka, Japan; 5 Department of Neurology, National Hospital Organization Toneyama Hospital, Toyonaka, Osaka, Japan; 6 Department of Neurology, National Hospital Organization Hyogo-Chuo Hospital, Ohara, Hyogo, Japan; 7 Department of Molecular, Cell and Developmental Biology, RNA Center, Sinsheimer Labs, University of California Santa Cruz, Santa Cruz, California, United States of America; University of Edinburgh, United Kingdom

## Abstract

Myotonic dystrophy type 1 (DM1) is a multi-systemic disorder caused by a CTG trinucleotide repeat expansion (CTG^exp^) in the *DMPK* gene. In skeletal muscle, nuclear sequestration of the alternative splicing factor muscleblind-like 1 (MBNL1) explains the majority of the alternative splicing defects observed in the *HSA* ^LR^ transgenic mouse model which expresses a pathogenic range CTG^exp^. In the present study, we addressed the possibility that MBNL1 sequestration by CUG^exp^ RNA also contributes to splicing defects in the mammalian brain. We examined RNA from the brains of homozygous *Mbnl1* ^ΔE3/ΔE3^ knockout mice using splicing-sensitive microarrays. We used RT-PCR to validate a subset of alternative cassette exons identified by microarray analysis with brain tissues from *Mbnl1* ^ΔE3/ΔE3^ knockout mice and post-mortem DM1 patients. Surprisingly, splicing-sensitive microarray analysis of *Mbnl1* ^ΔE3/ΔE3^ brains yielded only 14 candidates for mis-spliced exons. While we confirmed that several of these splicing events are perturbed in both *Mbnl1* knockout and DM1 brains, the extent of splicing mis-regulation in the mouse model was significantly less than observed in DM1. Additionally, several alternative exons, including *Grin1* exon 4, *App* exon 7 and *Mapt* exons 3 and 9, which have previously been reported to be aberrantly spliced in human DM1 brain, were spliced normally in the *Mbnl1* knockout brain. The sequestration of MBNL1 by CUG^exp^ RNA results in some of the aberrant splicing events in the DM1 brain. However, we conclude that other factors, possibly other MBNL proteins, likely contribute to splicing mis-regulation in the DM1 brain.

## Introduction

Myotonic dystrophy type1 (DM1) is a multi-systemic disorder affecting skeletal muscle, heart, ocular lens, testis, and the central nervous system (CNS). CNS involvement in adult-onset DM1 includes visual spatial and attention deficits, dysexecutive syndrome, apathy, avoidant behavior and excessive daytime sleepiness (review [1]). Although neuropathological studies have revealed several morphological changes [2], [3], [4], whether these changes contribute to the clinical symptoms observed in DM1 remains to be determined.

DM1 is caused by the unstable expansion of CTG trinucleotide repeats in the 3′ untranslated region of the DM protein kinase (*DMPK*) gene [5]. Recent evidence suggests that transcripts containing expanded CUG repeats (CUG^exp^) accumulate in nuclear RNA foci and exert toxic effects on a variety of cellular regulatory pathways including splicing and transcription [6]. One disease model that is supported by considerable experimental evidence proposes that CUG^exp^ RNAs cause sequestration and inhibition of the RNA-binding protein MBNL1 [7]. In support of this model, *Mbnl1* knockout mice develop the muscle, eye, and RNA splicing abnormalities that are characteristic of DM1 disease [8]. In addition, we have reported a strong correlation in skeletal muscle splicing changes between two mouse models of DM1, the *HSA* ^LR^ transgenic and the *Mbnl1* knockout [9].

In the CNS of DM1 patients, mutant *DMPK* transcripts accumulate in neuronal nuclei and sequester MBNL1 and MBNL2 [10]. Although abnormal regulation of several alternatively spliced exons has also been documented in the DM1 brain [10], [11], [12], [13], it is not clear if MBNL1 sequestration contributes to aberrant splicing. Here we used splicing-sensitive microarrays to detect mis-splicing in the *Mbnl1* ^ΔE3/ΔE3^ knockout brain.

## Results

### Splicing perturbations in the *Mbnl1* ^ΔE3/ΔE3^ knockout brain

**Figure 1:**
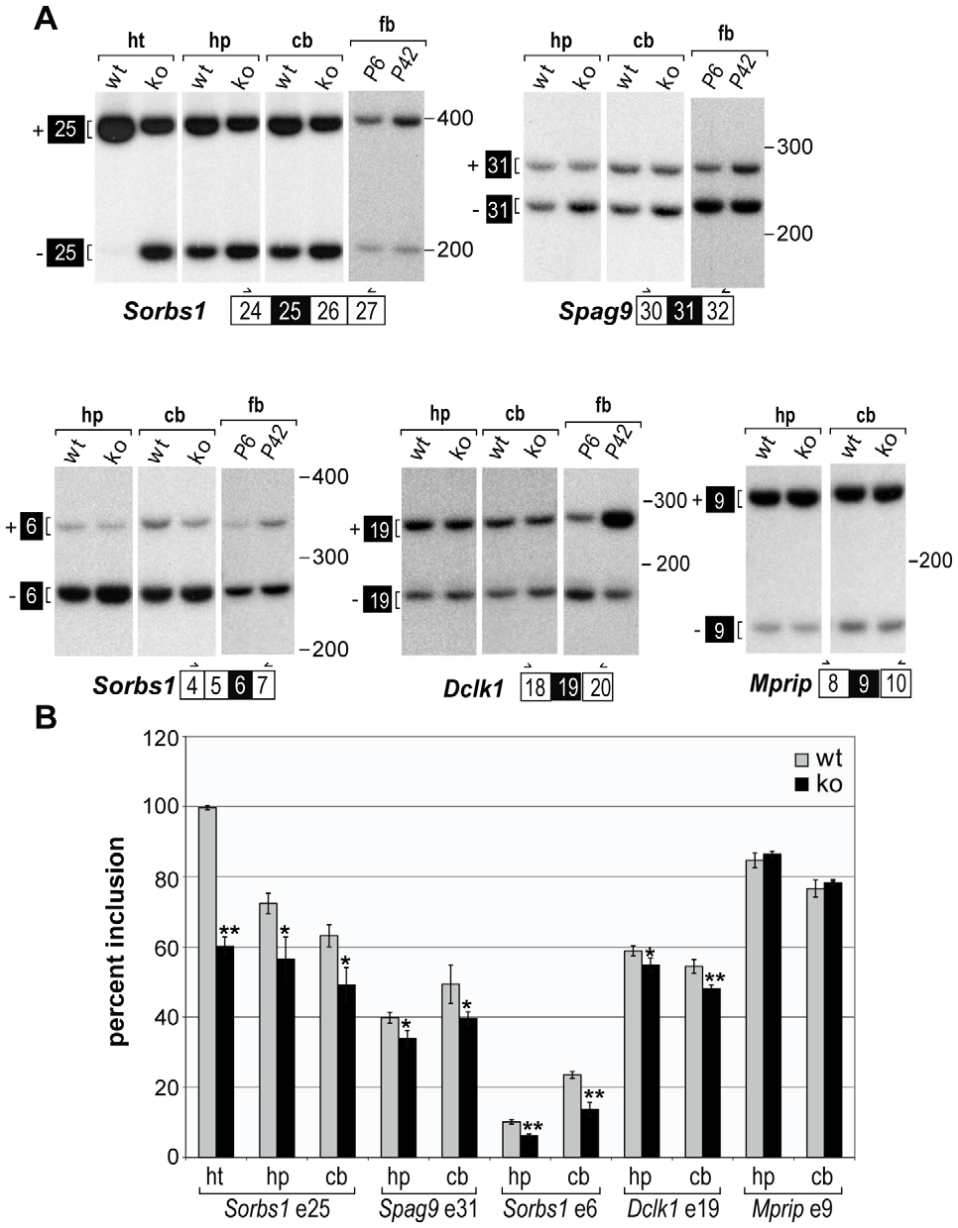
MBNL1 splicing targets in the mouse brain. RT-PCR analysis of wild-type (wt, n = 3, 3–6 months of age) versus *Mbnl1* ^ΔE3/ΔE3^ (ko, n = 3, 3–6months of age) brain splicing. (*A*) Representative PCR products. (*B*) Percent exon inclusion measured by phosphoimager analysis. Loss of MBNL1 expression results in reduced exon inclusion for *Sorbs1* exon 25, *Spag9* exon 31, *Sorbs1* exon 6 and *Dclk1* exon 19 in both the hippocampus (hp) and cerebellum (cb). The inclusion of these exons is enhanced in the adult (P42) forebrain (fb) compared to the neonatal (P6) forebrain. *Sorbs1* exon 25 splicing in the heart (ht) and *Mprip* splicing in the brain are included as positive and negative splicing controls. Both unpaired *t*-test and permutation test were used for calculating *p* value (*p value<0.05, **p value<0.01). Averages and standard deviations were generated by unpaired *t*-test.

To test directly whether loss of MBNL1 expression contributes to aberrant splicing in the CNS, RNA was extracted from the brains of age-matched males carrying homozygous *Mbnl1* ^ΔE3/ΔE3^ knockout or wild-type alleles in the same (C57BL/6J) background and analyzed on splicing-sensitive microarrays [9]. Experience with this method indicates that differences in the log_2_ of the ratio of exon skipping to inclusion (the skip/include ratio) between two samples (sepscore) with an absolute value >0.3 can be validated by RT-PCR. We observed 14 events in *Mbnl1* ^ΔE3/ΔE3^ knockout mice that exceeded this score (Table S1). We used RT-PCR to validate a subset of alternative cassette exons identified by microarray analysis and found four events (*Sorbs1* exons 6 and 25, *Spag9* exon 31, *Dclk1* exon 19) that showed significant differences in splicing between wild-type and knockout mice in both the hippocampus and cerebellum (Figure 1A,B). *Sorbs1* is particularly interesting because it is expressed in multiple tissues and exon 25 is regulated during postnatal development [9], [14]. In heart, MBNL1 is a major factor which regulates exon 25 splicing with nearly complete inclusion in wild-type adult heart but only ∼60% inclusion in the *Mbnl1* knockout heart. While this exon is also mis-spliced in the *Mbnl1* knockout brain, the effect of MBNL1 loss on this exon is considerably less in both the hippocampus and cerebellum. This modest effect of MBNL1 loss on other mis-regulated exons was also apparent for *Spag9* exon 31, *Sorbs1* exon 6 and *Dclk1* exon 19 (Figure 1B). Nevertheless, these exons are regulated during developmental transition from neonatal (P6) to adult (P42) forebrain (Figure 1A), which indicates other factor may play a role in splicing regulation during this period.

**Figure 2:**
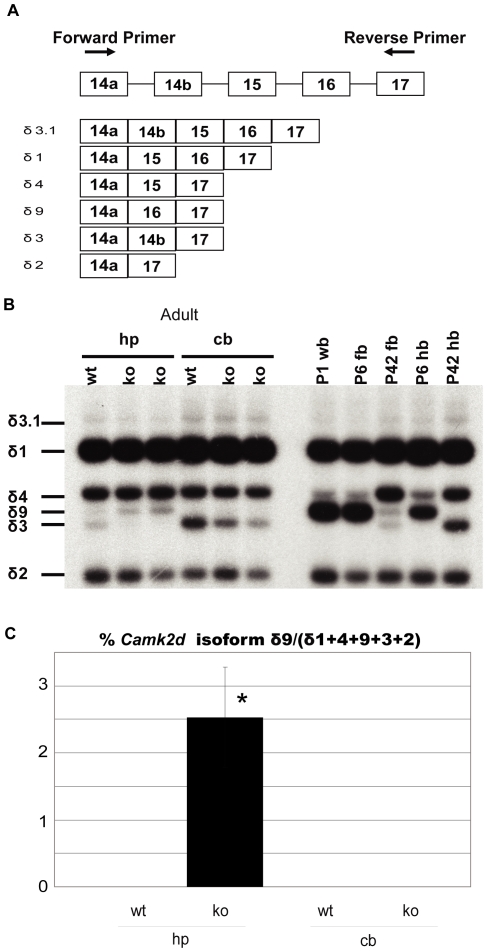
Spatial and temporal differences in the expression of *Camk2d*. (A) Transcripts of Camk2d. (B) Representative PCR products. Six spliced isoforms were detected by using primer pairs located in exons 14a and 17. Right panel shows isoform switching from δ9 to δ4 during the fetal (P1, P6) to adult (P42) transition in whole brain (wb), forebrain (fb) and hindbrain (hb) whereas the δ9 isoform was undetectable in adult hindbrain. Left panel shows a detectable increase in the δ9 isoform in *Mbnl1* ^ΔE3/ΔE3^ hippocampus (hp) compared with wild-type sibs. However, no δ9 isoform could be detected in the cerebellum of either wild-type or *Mbnl1* knockout mice. (C) The percentage of *Camk2d* isoform δ9 to δ1+4+9+3+2. Both unpaired *t*-test and permutation test were used for calculating *p* value (n = 3, *p value<0.05). Averages and standard deviations were generated by unpaired *t*-test.

Splicing microarrays also revealed mis-splicing of *Camk2d*, so we further investigated splicing of this gene. Importantly, *Camk2d* splicing is regulated during postnatal development with the major isoforms all containing exons 14a and 17 with alternative splicing of the exon 14b, 15 and 16 cassettes to generate several major isoforms, including δ1, δ2, δ3, δ3.1, δ4 and δ9 (Figure 2). Previous reports have indicated that the δ1 isoform is the major splice variant in the adult rat forebrain, while δ4 isoform is expressed in the neonatal rat forebrain [15]. We observed that in P1 whole brain, P6 fore- and hindbrain, δ1 and δ9 are the major isoforms while in adults (P42), exon 15 is preferentially spliced, so that in addition to δ1, δ4 is also the major form in forebrain and both δ4 and δ3 are expressed in hindbrain. In accordance with other splicing changes where immature splicing isoforms are expressed in the adult tissues, splicing of the fetal δ9 isoform significantly increased in the hippocampus of *Mbnl1* knockouts compared to controls although the increase was modest and was not observed in the cerebellum. While the δ9 isoform increased, the δ3 (+14b −15 −16) isoform decreased in *Mbnl1* knockout mouse hippocampus compared with wild-type.

### Aberrant splicing in human brain samples and comparison with *Mbnl1* knockout brain

**Figure 3:**
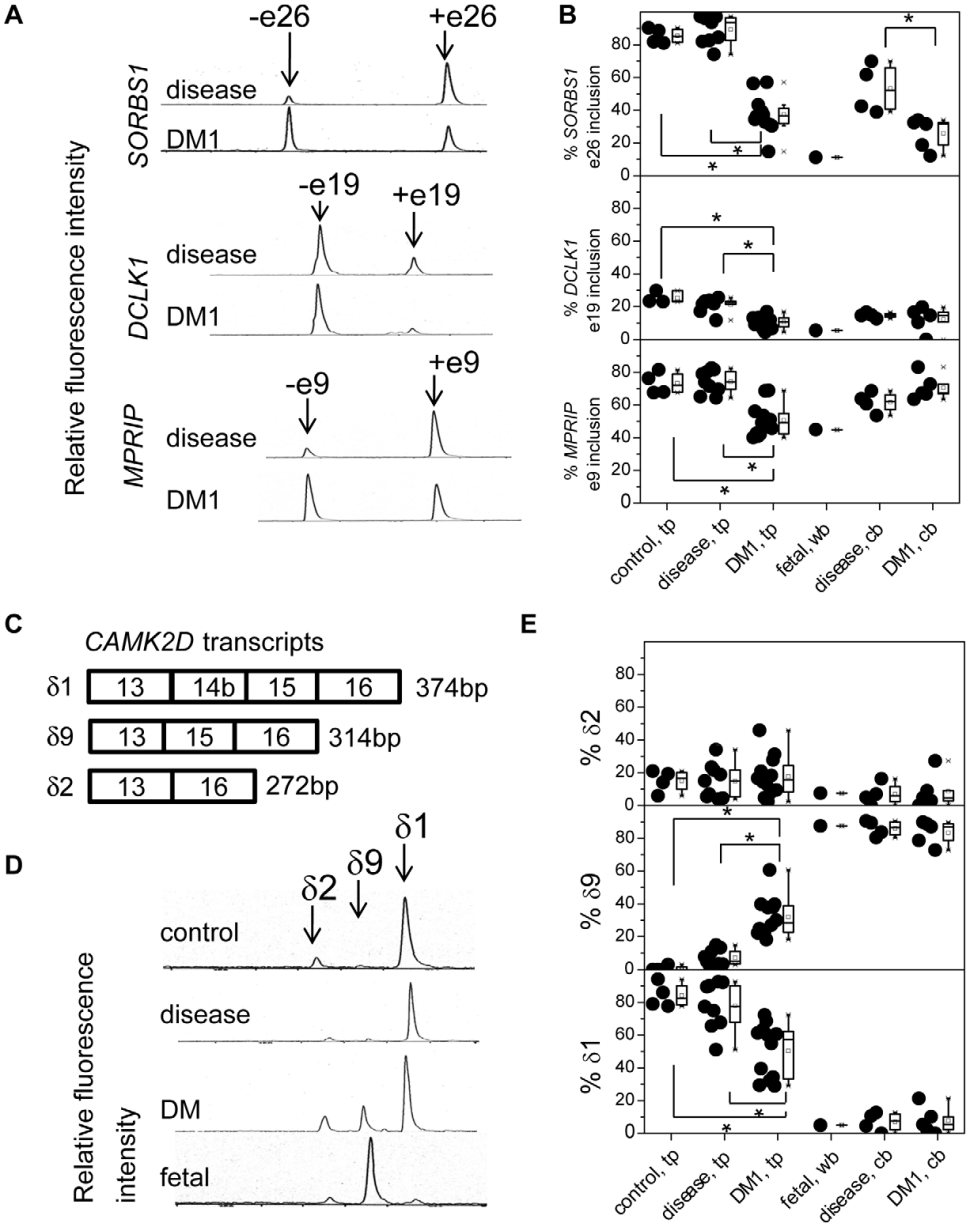
Aberrant splicing events in *Mbnl1* ^ΔE3/ΔE3^ brain are also observed in the human DM1 brain. We compared brain RNA from normal control temporal cortex (control, tp, n = 4), disease control temporal cortex (disease, tp, n = 9), myotonic dystrophy type 1 temporal cortex (DM1, tp, n = 12), fetal control whole brain (fetal, wb, n = 1), disease control cerebellum (disease, cb, n = 4), and DM1 cerebellum (DM1, cb, n = 5). (A) Representative RT-PCR products detected with microchip electrophoretic separation using SV1210 software on the Hitachi SV1210 from exclusion and inclusion of exon 26 of *SORBS1* (upper), exon 19 of *DCLK1* (middle), and exon 9 of *MPRIP* (bottom). (B) Graphical representation of RT-PCR analysis depicting percentages of exon 26 inclusion of *SORBS1* (upper), exon 19 inclusion of *DCLK1* (middle), and exon 9 inclusion of *MPRIP* (bottom). (C) Transcripts of *CAMK2D*. (D) Representative RT-PCR products from *CAMK2D* isoforms: δ2(−exon14b−exon15), δ9(−exon14b+exon15), and δ1(+exon14b+exon15) indicated by each arrow. (E) Graphical representation of RT-PCR analysis depicting percentages of δ2 (upper), δ9 (middle) and δ1 (bottom). Mann-Whitney U test was used for calculating the *p* value. Statistically significant differences (*p*<0.05) are indicated by an asterisk.

To determine whether these mis-splicing events predicted from the *Mbnl1* knockout brain microarray data were also observed in the human DM1 brain, we aligned affected mouse exons to the human genome and found 12 exons that are conserved and orthologous to MBNL1-dependent mouse exons (Table S2). We examined all of these events in post-mortem human temporal cortex and cerebellum. The most striking result in human DM1 brain was mis-splicing of *SORBS1* exon 26 (orthologous to mouse exon 25). In the temporal cortex, the average percentage of exon 26 inclusion in DM1 was ∼40% while those observed in the normal controls and in the disease controls were ∼85% and ∼90%, respectively (Figure 3A and B, upper). In the cerebellum, exon 26 inclusion was also significantly decreased in DM1 compared with in disease controls, although this difference is subtler than that in the temporal cortex. Both *DCLK1* exon 19 inclusion and *MPRIP* exon 9 inclusion were significantly decreased in DM1 temporal cortex (Figure 3A and B, middle and bottom), whereas there is no difference in these splicing events between DM1 and disease cerebellums.

In humans, the splicing of *CAMK2D* is also regulated in a tissue-specific and developmental manner. We found that δ9 was expressed almost exclusively in human fetal whole brain (unlike rodent brain), while δ1 was predominantly expressed in adult human temporal cortex samples (Figure 3C to E). In contrast, the percentage of the δ9 isoform was significantly increased in adult DM1 temporal cortex compared to both disease and normal controls. This increase was ∼30% in DM1 temporal cortex (Figure 3E), or apparently larger than that observed in the hippocampus of *Mbnl1* knockout mice (Figure 2), although we could not conclude which of the following changes will have a stronger impact: (a) an increase from 0 to ∼2.5% in *Mbnl1* knockout mice (Figure 2C), or (b) an increase from ∼10% to ∼30% in DM1 temporal cortex (Figure 3E). As a result of the increase of δ9, the percentage of δ1 was significantly decreased in the DM1 temporal cortex. In DM1 and disease control cerebellums, δ9 was a major isoform and there is no difference between these two. Unlike mouse cerebellum, δ3 isoform was not observed in any groups of human brains.

Initially we did temporal lobe autopsy on human since a former report showed splicing defects of several exons in this region [10]. We did hippocampus analysis on mouse because it is a key region of the brain involved in learning and memory. We thought a concern of regional difference between hippocampus and temporal lobe may not be an issue since a report showed similar tau pathology in hippocampus and temporal cortex [4] and we have compared the splicing pattern of each exon in these regions of mouse brain and found similar splicing defects of *Sorbs1* exon 25 and *Camk2d* exon 14–16 (increased expression of δ9) in *Mbnl1* knockout mice (Figure S1). We also used cerebellum samples as a reference region since no apparent cerebellar symptoms in DM1 patients and the lack of RNA foci in DM1 cerebellar cortex have been reported [10]. Although we did not show a direct binding of MBNL1 on these RNA by CLIP (cross linked immuno precipitation), we identified several YGCY motifs in the upstream and downstream introns of these alternative spliced cassettes (Table S3).

We determined by Southern blot analysis the CTG repeat number of the same brain tissues from which we took RNA for RT-PCR (Table 1). Cerebral cortex showed greater CTG repeat number and larger intra-tissue CTG length heterogeneities than cerebellum, consistent with a previous observation [16]. The comparison between the degree of aberrant splicing of each exon and each shortest, median and longest CTG repeat number showed no correlation (Figure S2). Several splicing defects have been reported in DM1 brain and we confirmed some of them (exon 4 of *GRIN1*, exons 3 and 12 of *MAPT* and exon 9 of *APP*) in our human DM1 temporal cortex samples (Figure S3A). However, our splicing microarray analysis did not detect any mis-splicing of these exons in the *Mbnl1* knockout model. RT-PCR using each set of specific primer for these exons (*Grin1* exon 4, *Mapt* exons 3 and 9, and *App* exon 7) also revealed no defects of splicing (Figure S3B).

**Table 1:** Human samples analysed.

Patient no/sex	Age	disaese	Sources	CTG repeats in brain samples
C1/M	23	anormal	temporal cortex	
C2/M	22	anormal	temporal cortex	
C3/M	26	anormal	temporal cortex	
C4/F	78	bnormal	temporal cortex	
D1/F	69	PD	temporal cortex	
D2/F	84	MSA-C	temporal cortex	
D3/F	80	ALS	temporal cortex	
D4/M	53	ALS	temporal cortex	
			cerebellum	
D5/F	81	ALS	temporal cortex	
			cerebellum	
D6/M	70	ALS	temporal cortex	
D7/F	75	ALS	temporal cortex	
D8/F	61	ALS	temporal cortex	
			cerebellum	
D9/M	70	ALS	temporal cortex	
			cerebellum	
DM1/M	58	DM1	temporal cortex	3000–6000
DM2/M	69	DM1	temporal cortex	3000–6000
DM3/M	50	DM1	temporal cortex	2000–6000
DM4/F	56	DM1	temporal cortex	3000–5000
			cerebellum	400
DM5/M	63	DM1	temporal cortex	1200–3000
DM6/M	59	DM1	temporal cortex	3000–9000
DM7/F	58	DM1	temporal cortex	3000–6000
			cerebellum	2200–3000
DM8/F	58	DM1	temporal cortex	2000–8000
DM9/F	58	DM1	temporal cortex	2200–6500
			cerebellum	300
DM10/F	66	DM1	temporal cortex	1900–3300
			cerebellum	150
DM11/M	64	DM1	temporal cortex	2400–5200
DM12/F	73	DM1	temporal cortex	1900–4800
			cerebellum	200
F1/F	21w	cfetal	whole brain	

a from Biochain,

b from Ambion,

c from Stratagene, PD; Parkinson's disease; MSA-C; multiple system atrophy with predominant cerebellar ataxia; ALS: amyotrophic lateral sclerosis.

## Discussion

In the present study, we found three novel splicing events, *Sorbs1* exon 25 (exon 26 in human), *Dclk1* exon 19, and *Camk2d* exon 14–16 (exon 14–15 in human), altered both in DM1 and in *Mbnl1* knockout brains. This is the first report of mis-splicing of orthologous exons in brain tissues from human DM1 and *Mbnl1* knockout mice, although mis-splicing of exons 3 and 12 (9 in mice) of *MAPT* has been shown in human DM1 brain [10], [13] and in the CTG transgenic mouse brain [17]. In general, the *Mbnl1* knockout hippocampus and DM1 temporal cortex shared similar splicing defects. We also confirmed similar splicing defects of some genes in the hippocampus and temporal cortex of *Mbnl1* knockout mice (Figure S1). The expression of the fetal splicing isoforms in our samples is consistent with other splicing abnormalities in which fetal splice isoforms are expressed in adult DM1 patients and model mice. Interestingly, only the increase in *GRIN1* exon 4 inclusion in DM1 brain does not represent a return to a fetal splicing profile (Figure S3), which may suggest that distinct splicing factors regulate this splicing event. Our data did not demonstrate directly that MBNL1 regulates the splicing of these exons. However, we found many YGCY motifs that could potentially serve as MBNL1 binding motifs (Table S3) [9].

In human cerebellum, aberrant splicing was less apparent and only splicing of *SORBS1* exon 26 was altered significantly, while similar splicing defects were shown in the hippocampus and cerebellum of the *Mbnl1* mouse knockout model. One possible reason for this modest splicing alteration in the cerebellum of human DM1 is that fetal splicing isoforms of some genes are already dominantly expressed in human adult cerebellum and thus there is no room for any detectable difference of fetal isoforms between DM1 and control samples (e.g. *CAMK2D* in which fetal δ9 isoform is almost exclusively expressed in both disease control and DM1 cerebellums relative to fetal brain (Figure 3E)). Another possibility is that MBNL1 may not be as efficiently sequestered in RNA foci in the DM1 cerebellar nucleus because the CTG repeat expansion is much smaller in the cerebellum than in the temporal cortex (Table 1). One alternative possibility is that MBNL1 contributes less significantly to the regulation of splicing in human cerebellum. This possibility is unlikely, however, for the following reasons. First, both the immunohistochemistry and western blotting displayed similar expression of MBNL1 in different regions of the brain (Figure S4, Material and Methods S1). Second, since our RT-PCR demonstrated similar splicing defects in Mbnl1 targeted exons in both the hippocampus and cerebellum of Mbnl1 knockout mice (Figure 1), it seems that the contributions of Mbnl1 to these regions are similar.

Compared with *Mbnl1* knockout skeletal muscle, where 246 events achieved sepscore values of >0.3 [9], we observed far fewer splicing events that exceeded this score. Some exons previously reported to be mis-spliced in the DM1 brain were not observed in the *Mbnl1* knockout brain. In addition, the extent of splicing mis-regulation in the mouse model was significantly less than observed for DM1 (Figures 1, 2, 3). A possible explanation for these smaller splicing changes in *Mbnl1* knockout mouse brain is that there are species differences of splicing regulation between mouse and human. However, transgenic mouse brains carrying over 700 CTG repeat have been reported to display splicing abnormalities of *Mapt*, *Grin1*, *Mbnl1*, and *Mbnl2* [17]. These results suggest that the contribution of MBNL1 to developmental splicing transitions in the brain is less significant than in skeletal muscle or that the cellular complexity of the brain masks a larger effect of MBNL1 loss on particular cell populations. Three genes encode *MBNL* paralogs in mammals, *MBNL1*, *MBNL2* and *MBNL3*, and all of the encoded proteins have been demonstrated to colocalize with nuclear foci in DM1 cells [18]. In reconstruction experiments using transfected cells, each MBNL protein can regulate alternative splicing of pre-mRNAs that are misregulated in DM1 [19]. As MBNL3 is expressed predominantly in the placenta [18], the likely candidate which may play a more significant role in alternative splicing in the brain is MBNL2. While human MBNL1 is expressed primarily in skeletal muscle and heart, MBNL2 is expressed at a similar level in the majority of tissues [18]. Thus, the contribution of MBNL2 to splicing changes may be relatively high in brain in contrast to skeletal muscle. It has been reported that *Mbnl1* knockout mice demonstrate cognitive and behavioral abnormalities and normal spatial learning and memory [20]. This relatively mild phenotype may correlate with the modest splicing alteration. Regarding exon 31 of *Spag9* and exon 6 of *Sorbs1*, we found the significant difference between wild-type and *Mbnl1* knockout, but not in human brains. This discrepancy may contradict our hypothesis that the recruitment of MBNL1 contributes to the splicing defects in DM1 brains, but may be explained by a difference of splicing regulation between mice and human.

In vitro experiments suggested that a loss of MBNL1 or a gain of CELF1 function could repress exon 3 inclusion but not exon 9 inclusion of *Mapt*, whereas CELF2 is a repressor of both exons [21]. However, *Mbnl1* knockout mice did not show any splicing differences for these exons. This discrepancy about MBNL1 dependent regulation of the splicing of *Mapt* exon3 between in vitro and mice experiments may also indicate that some redundant factors function in this splicing regulation and loss of MBNL1 alone is insufficient to change the splicing pattern of this exon in the brain.

SORBS1 was identified as a Cbl-associated protein likely to participate in insulin signaling [22]. The targeted exon encodes the nuclear localization sequence (NLS) [23], suggesting that the protein without an NLS is increased in the DM1 brain and might not be able to function as a nuclear protein. Among the four types of CAMK2 isoforms, the CAMK2D is expressed predominantly in the suprachiasmatic nucleus, substantia nigra, and striatum in the brain. Since each spliced isoform of CAMK2D has been reported to show a distinct distribution pattern [24], [25], the increase in δ9 might cause abnormal localization. Interestingly, CAMK2D has been reported to play an essential role in PER1 expression, which is possibly involved in light-induced phase shifts [26]. Although excessive daytime sleepiness and dysregulation of REM sleep occur frequently in patients with DM1, it was reported that the pathophysiologic basis is distinct from narcolepsy, as patients with DM1 do not have a consistent defect of hypocretin release or receptor splicing [27]. Possibly, aberrant splicing of *CAMK2D* might be associated with excessive daytime sleepiness in DM1. DCLK1 is a microtubule-associated kinase that can undergo autophosphorylation and regulate microtubule polymerization [28]. The fetus specific C-terminal splicing variant (exon 19 exclusion), which is expressed more in DM1 adult temporal cortex, showed enhanced autophosphorylation activity and has been suggested a possible role in migrating neurons [29]. Therefore, the increase in the fetal variant of DCLK1 in DM1 brain may cause an abnormal neuronal migration. MPRIP was initially identified as an 116 kDa protein that interacts with RhoA [30]. In neuronal cells, MPRIP is essential for neurite outgrowth and may act as a scaffold to target the myosin phosphatase complex to the actin cytoskeleton [31]. Although the difference between splicing isoforms has not been reported, aberrant splicing of this gene could give rise to abnormal neurite outgrowth in DM1 brain.

In this study, we detected three novel splicing targets which were mis-regulated in both *Mbnl1* knockout mice and human DM1, two only in *Mbnl1* knockout mice, and one only in human DM1 brains. Since the brain is a heterogeneous organ consisting of many different cell types, splicing events may be regulated in a region-specific manner. Our studies support this interpretation with distinct variations in the splicing of developmentally-regulated exons observed in the hippocampus and cerebellum. Our results suggest that at least some of the aberrant splicing events in the DM1 brain result from the sequestration of MBNL1 by CUG^exp^ RNAs. Additionally, it is also likely that other RNA-associated proteins contribute to the aberrant splicing in DM1. Thus, it will be important to investigate the splicing targets in brain samples derived from other DM1 model mice, including *Celf1* transgenic, *Celf2* transgenic and *Mbnl2* knockout mice.

## Materials and Methods

### Ethics Statement

All experiments using mice were performed in accordance with the guidelines of the University of Florida. The protocol was approved by the University of Florida Institutional Animal Care and Use Committee (Permit Number: #200903677). All experiments using human samples were approved by the Ethics Committee of Hyogo College of Medicine (Permit number: 93) and written informed consent for specimen use for research was obtained from all patients.

### Splicing Microarray Detection

RNA samples from the brain of individual 12–14-week-old male mice from the wild-type and *Mbnl1* ^ΔE3/ΔE3^ lines (n = 4 for each group) were compared. To identify MBNL1-dependent splicing events, RNA from *Mbnl1* ^ΔE3/ΔE3^ mice was compared to wild-type mice in the C57BL/6J (N10) background. RNA samples were processed for cDNA synthesis and hybridization to Affymetrix “A-chip” oligonucleotide microarrays as follows. Total RNA was primed with random hexamers and reverse transcribed. After the reaction was completed, RNA was removed from the reaction by alkaline hydrolysis and the cDNA was purified using Qiagen PCR Quick Purification Kit. A typical reaction started with 5 µg of total RNA usually yielded ∼3 µg of cDNA. The cDNA was then fragmented using DNase I in an empirically controlled reaction that yields DNA fragments of 50–200 bases. This fragmented cDNA was then end labeled using terminal deoxynucleotidyl transferase and “DNA-Labeling-Reagent-1a (DLR-1a)”, which is a biotinylated dideoxynucleoside triphosphate. Labeled cDNA was hybridized to arrays in standard Affymetrix hybridization buffer for 16 hrs at 50°C. Arrays were washed, processed with anti-biotin antibodies and streptavidin-phycoerythrin according to the standard Affymetrix protocol.

### Microarray Data Analysis

Analysis was done according to Sugnet et al. [32], who calculate a “separation score” that measures the relative change in ratio of alternative splicing events. The equation for sepscore is:
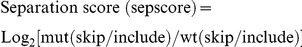
For each replicate set (each tissue from *Mbnl1* ^ΔE3/ΔE3^ and wild type), we estimated the log_2_ ratio of skipping to inclusion using robust least squares analysis. We evaluated sepscore significance by permuting the assignments of data points to replicate sets, calculating the separation score for the permuted data, and estimating the likelihood that the observed data came from the permuted distribution. The microarray data have been deposited in NCBI's Gene Expression Omnibus and are accessible through GEO series accession number GSE28640 (http://www.ncbi.nlm.nih.gov/geo/query/acc.cgi?acc=GSE28640). All data is MIAME compliant and the raw data has been deposited in a MIAME compliant database (GEO), as detailed on the MGED Society website (http://www.mged.org/Workgroups/MIAME/miame.html).

### Target validation by RT-PCR for model mice

The sequences of primers used for RT–PCR assays of splicing in mouse and human brain RNA are listed in (Tables S1 and S2). To test mouse MBNL1 potential splicing targets identified by splicing microarrays, we harvested hippocampal, temporal cortex and cerebellar tissues from *Mbnl1* ^+/+^ (n = 3) and *Mbnl1* ^ΔE3/ΔE3^ (n = 3) mice (C57BL/6J background) ranging from 3–6 months of age. RNA was isolated using TRI Reagent (Sigma) and cDNA generated using 5 µg of RNA, oligo (dT)_20_ (Invitrogen) and SuperScript III (Invitrogen) followed by digestion with 0.2 units/µl RNase H (Invitrogen) at 37°C for 20 min. Splicing targets with predicted alternately spliced cassettes were selected from microarray list and 5 ng of cDNA was PCR-amplified for 28 cycles (95°C for 30 sec, 55°C for 30 sec, 72°C for 30 sec) by using forward and reverse primers located in the flanking exons. Each PCR reaction was spiked with 2.5 µCi of [α^32^P]-dCTP (PerkinElmer Life Sciences). PCR products were resolved on 10% non-denaturing polyacrylamide gels followed by autoradiography using Kodak BioMax MS film (PerkinElmer Life Sciences). Exon inclusion was measured using a Typhoon 9200 imager and ImageQuant TL software (GE Healthcare).

### RT-PCR and Southern blot analysis for human brain samples

Human temporal cortex and cerebellar tissues (n = 21) at autopsy were analyzed (Table 1). RNA was extracted from each sample by the ISOGEN procedure (Nippon Gene). The RNAs used as normal controls were purchased commercially as follows: human adult temporal cortex (3 male, 1female, 3 samples from Biochain, 1 sample from Ambion) and 1 sample of human fetal whole brain (Stratagene) (Table 1). The quality of RNA and the PCR product were analyzed by capillary electrophoresis using a Hitachi SV 1210 on a microchip (Hitachi Electronics Co., Tokyo, Japan) [33]. We used the samples which meet the criteria that the ratio of 28S/18S rRNA peaks was >0.8. The CTG trinucleotide repeat expansion sizes of human brain samples were determined by Southern blot analysis of genomic DNAs from each brain region tissue of DM1 as described previously [34]. cDNA was synthesized using the same method as for mouse samples except 1–3 µg of RNA and random hexamers were used. cDNA equivalent to 20 ng RNA was PCR-amplified for initial denaturation at 94°C for 10 min and 35 cycles (94°C for 30 sec, 55°C for 30 sec, 72°C for 30 sec) by using forward and reverse primers located in the exons adjacent to the human exons orthologous to mouse exons predicted by the microarray data (Table S2). We determined the percentage of each peak by dividing each signal by the total signal.

### Statistical analysis

Differences for the levels of exon inclusion for age-matched genetically homogeneous mice were analyzed by unpaired t- and permutation tests. The data for human samples were analyzed using the nonparametric Mann-Whitney U test because the backgrounds such as age and CTG repeat number of each sample are regarded inhomogeneous and the data for these samples were not always normally distributed.

## Supporting Information

Figure S1 ***Sorbs1*** **exon 25 inclusion is decreased and** ***Camk2d*** **δ9 is increased to similar extent in the hippocampus (hp) and temporal cortex (tp) of** ***Mbnl1*** **^ΔE3/ΔE3^ (ko) mice compared with wild type (wt) mice.**
(TIF)

Figure S2 **Comparison between the degree of aberrant splicing and CTG repeat number in DM1 brain.** There was no significant correlation between each shortest, median and longest CTG repeat number, and percent inclusion of each of the exons which are mis-regulated. (A) Temporal tissues. (B) Cerebellar tissues. Spearman rank correlation coefficient was used for the analysis of correlation.
(TIF)

Figure S3 **Some splicing defects which have been reported in DM1 brain were not reproduced in** ***Mbnl1*** **knockout brain.** (A) Four splicing exons are mis-regulated in our human DM1 temporal cortex (exon4 of *GRIN1*, exons 3 and 12 of *MAPT*, and exon9 of *APP*). Mann-Whitney U test was used for calculating the *p* value. Statistically significant differences (*p*<0.05) are indicated by an asterisk. (B) These exons are normally spliced in the hippocampus (hp) of *Mbnl1* knockout mice.
(TIF)

Figure S4 **MBNL1 distribution and expression in regions of mouse brain.** (A) Immnunohistochemistry with anti-MBNL1 antibody shows that MBNL1 is ubiquitously expressed in the frontal cortex (top), hippocampus (middle), and cerebellum (bottom) sections from wild-type (wt, right) but not in those from *Mbnl1* knockout (ko, left). (B) Western blot analysis shows a similar expression of MBNL1 in the cerebellum, temporal cortex, and hippocampus of a wild-type mouse. (Materials and Methods S1).
(TIF)

Materials and Methods S1 **Supplementary Materials and Methods for Muscleblind-Like 1 Knockout Mice Reveal Novel Splicing Defects in the Myotonic Dystrophy Brain.**
(DOC)

Table S1 **Separation score and primers for RT-PCR of mouse brain.**
(DOC)

Table S2 **Primers for RT-PCR of human brain.**
(DOC)

Table S3 **YGCY motif searching list with sequences exons aberrantly regulated in brains of** ***Mbnl1*** **knockout mice and DM1 patients.** YGCY motifs are indicated by capitals.
(DOC)
