# Supplementary figures and images for: Muscleblind-Like 1 Knockout Mice Reveal Novel Splicing Defects in the Myotonic Dystrophy Brain
Source: PLoS One. 2012 Mar 13;7(3):e33218. doi: 10.1371/journal.pone.0033218 (PMC3302840; doi:10.1371/journal.pone.0033218)

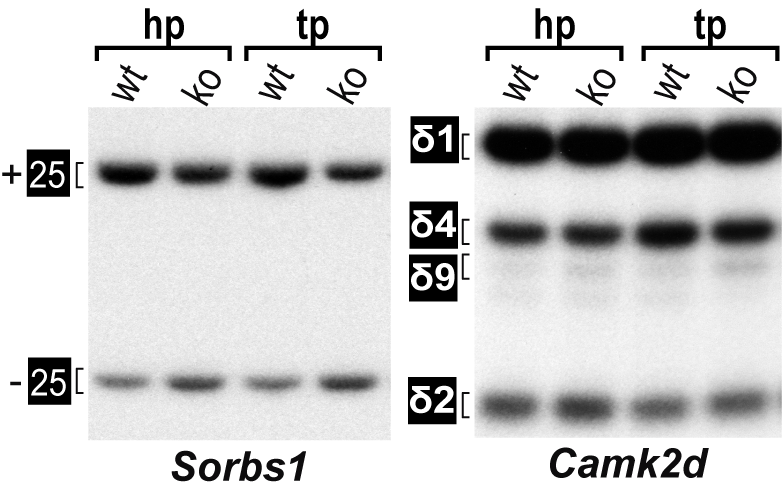

Supplement: Figure S1 — Sorbs1 exon 25 inclusion is decreased and Camk2d δ9 is increased to similar extent in the hippocampus (hp) and temporal cortex (tp) of Mbnl1 ΔE3/ΔE3 (ko) mice compared with wild type (wt) mice. (TIF) [file pone.0033218.s001.tif]

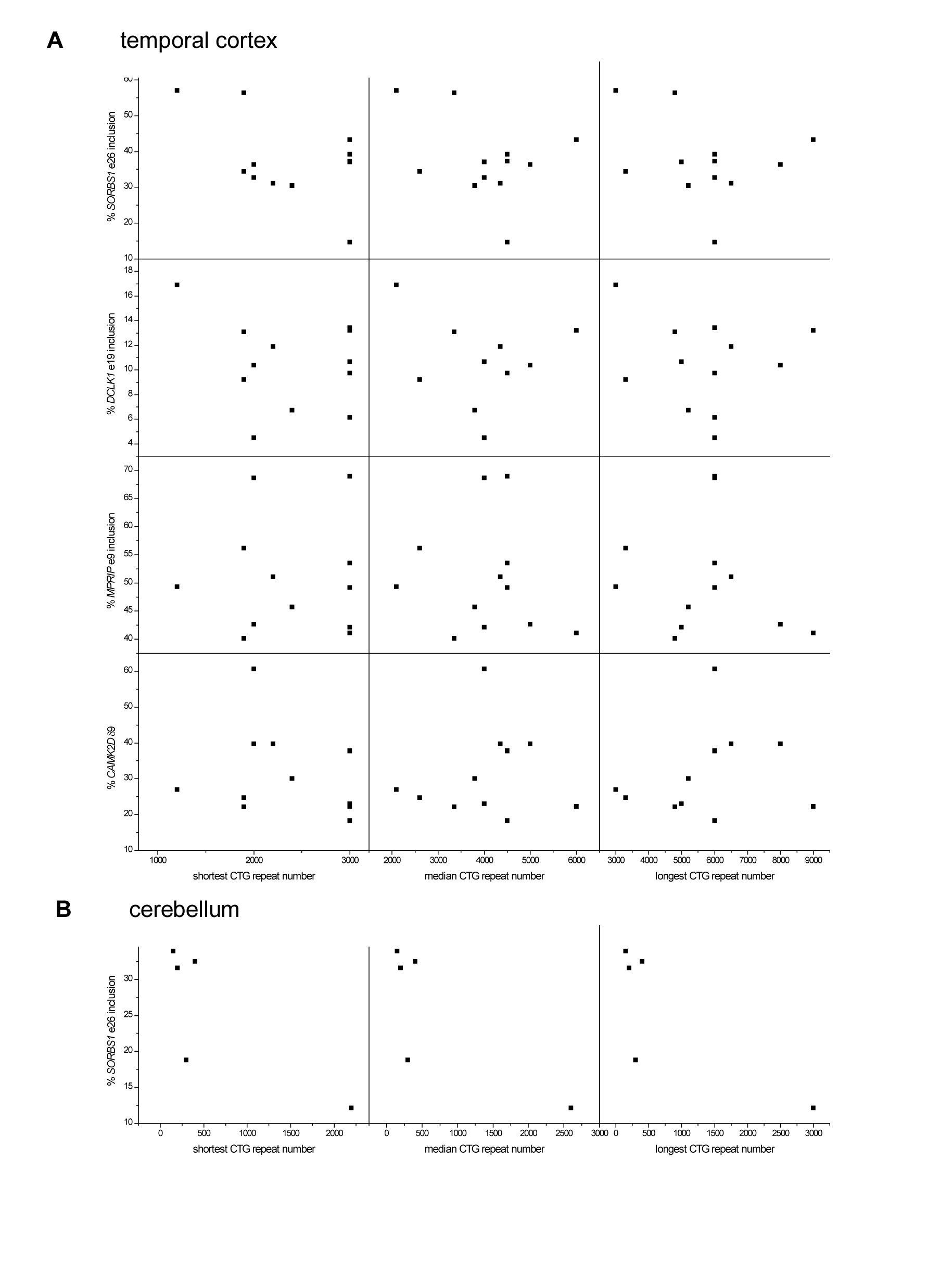

Supplement: Figure S2 — Comparison between the degree of aberrant splicing and CTG repeat number in DM1 brain. There was no significant correlation between each shortest, median and longest CTG repeat number, and percent inclusion of each of the exons which are mis-regulated. (A) Temporal tissues. (B) Cerebellar tissues. Spearman rank correlation coefficient was used for the analysis of correlation. (TIF) [file pone.0033218.s002.tif]

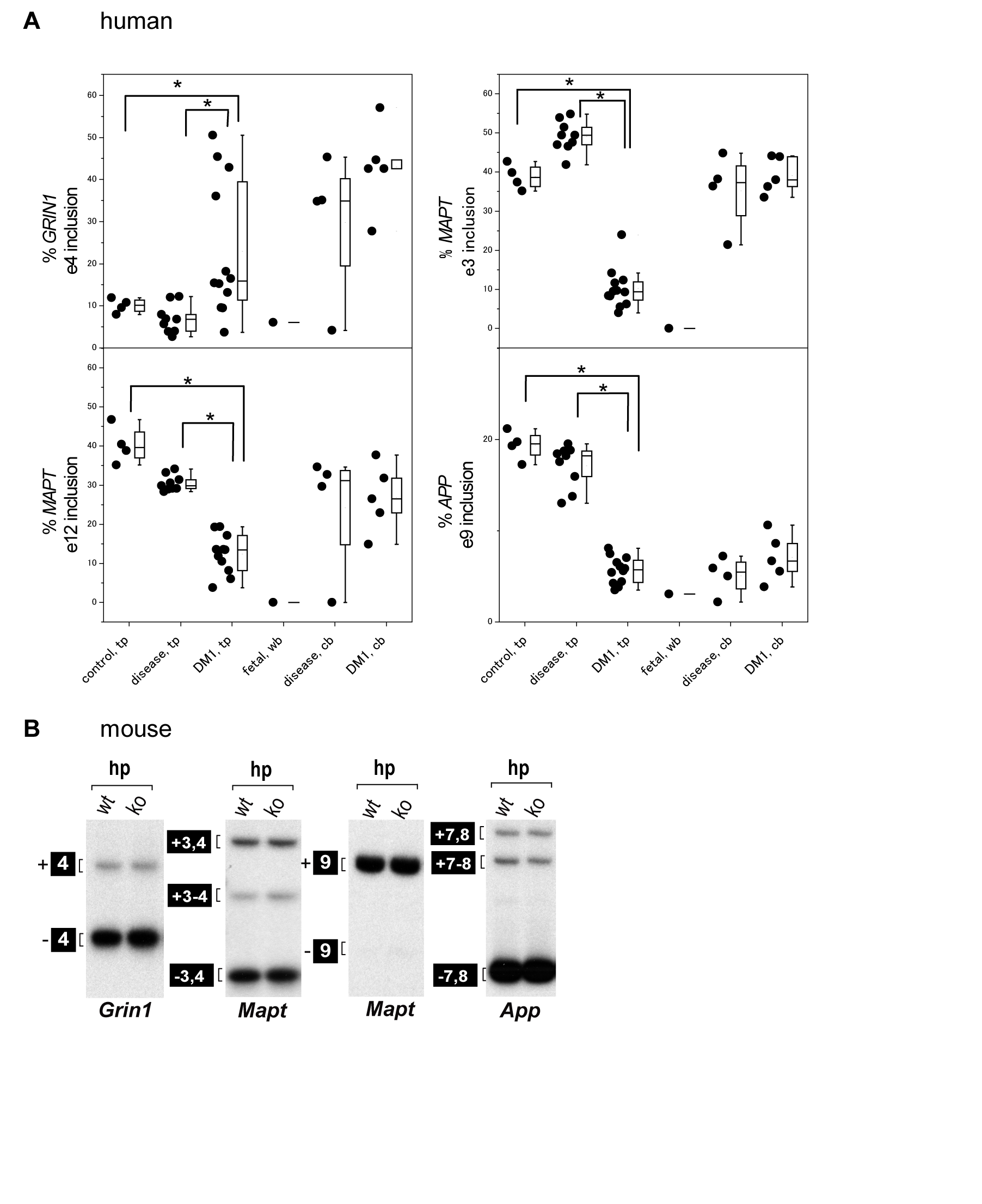

Supplement: Figure S3 — Some splicing defects which have been reported in DM1 brain were not reproduced in Mbnl1 knockout brain. (A) Four splicing exons are mis-regulated in our human DM1 temporal cortex (exon4 of GRIN1, exons 3 and 12 of MAPT, and exon9 of APP). Mann-Whitney U test was used for calculating the p value. Statistically significant differences (p<0.05) are indicated by an asterisk. (B) These exons are normally spliced in the hippocampus (hp) of Mbnl1 knockout mice. (TIF) [file pone.0033218.s003.tif]

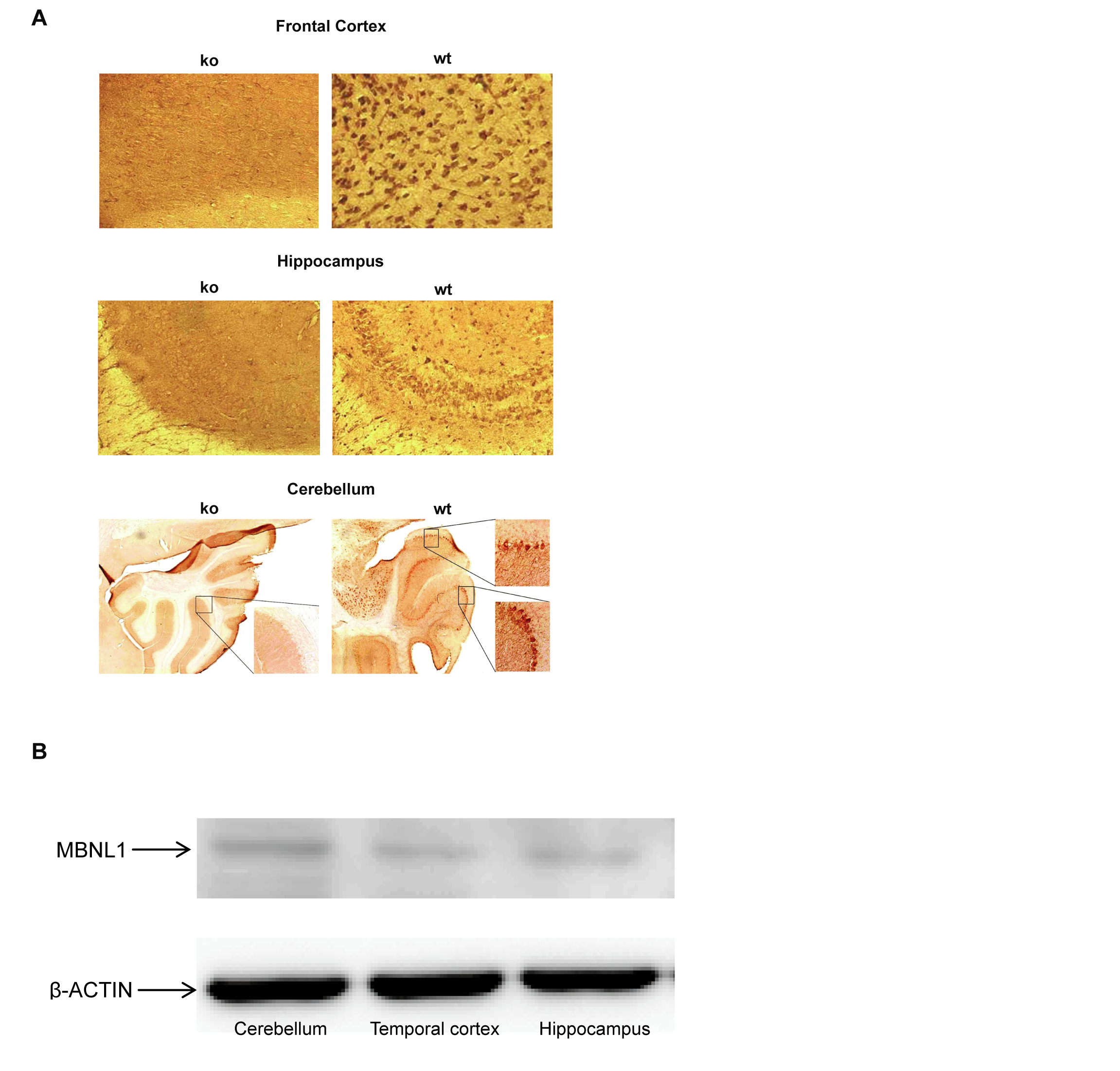

Supplement: Figure S4 — MBNL1 distribution and expression in regions of mouse brain. (A) Immnunohistochemistry with anti-MBNL1 antibody shows that MBNL1 is ubiquitously expressed in the frontal cortex (top), hippocampus (middle), and cerebellum (bottom) sections from wild-type (wt, right) but not in those from Mbnl1 knockout (ko, left). (B) Western blot analysis shows a similar expression of MBNL1 in the cerebellum, temporal cortex, and hippocampus of a wild-type mouse. (Materials and Methods S1). (TIF) [file pone.0033218.s004.tif]
