# Supplementary material for: Muscleblind-Like 1 Knockout Mice Reveal Novel Splicing Defects in the Myotonic Dystrophy Brain
Source: PLoS One. 2012 Mar 13;7(3):e33218. doi: 10.1371/journal.pone.0033218 (PMC3302840; doi:10.1371/journal.pone.0033218)
Supplement: Materials and Methods S1 — Supplementary Materials and Methods for Muscleblind-Like 1 Knockout Mice Reveal Novel Splicing Defects in the Myotonic Dystrophy Brain. (DOC) [file pone.0033218.s005.doc]

**Material and Methods S1**

Supplementary Materials and Methods for Muscleblind-Like 1 Knockout Mice Reveal Novel Splicing Defects in the Myotonic Dystrophy Brain.

Koichi Suenaga^1☯^, Kuang-Yung Lee^2,3☯^, Masayuki Nakamori^4¤a^ , Yoshiki Tatsumi^1^, Masanori P. Takahashi^4^, Harutoshi Fujimura^5^, Kenji Jinnai^6^, Hiroo Yoshikawa^1^, Hongqing Du^7¤b^, Manuel Ares Jr^7^, Maurice S. Swanson^2^, Takashi Kimura^1*^

## **Immunohistochemistry**

Either wild type or Mbnl1 knockout mice on C57BL/6J background were perfused with phosphate buffered saline(PBS) followed by 4% paraformaldehyde in PBS. Brains were postfixed in the same fixative for 24 h and immersed in 30% sucrose in 0.1 M PBS at 4°C. A series of 40-μm thick sections were made on a cryostat (Leica CM3050 S; Leica Microsystems, Nussloch GmbH, Germany) and stored in anti-freezing solution.

Peroxidase staining was performed based on the following protocol: sections were pre-incubated with 0.5% H_2_O, 10% methanol in PBS for 15 min and blocked with 5% normal goat serum for 1 h. Sections were incubated for 16 h at room temperature with MBNL1 antibody (A2764 rabbit polyclonal antibody). Incubation with secondary goat anti-rabbit biotinylated antibody for 2 h at room temperature was followed by incubation with avidin–biotin–peroxidase complex (ABC kit ; Vector Laboratories, Burlingame, CA, USA). Reactions were visualized by using 3,3 diaminobenzidine (DAB)(Sigma) as a chromagen.

## **Western blot analysis**

The brain tissue samples from cerebellum, temporal cortex, and hippocampus of a wild-type mouse on C57BL/6J background were dissected. The samples were homogenized at ratio of 1g of tissue per 10ml of ice-cold homogenization buffer containing 1% SDS, 10mM PB pH 7.4, 5mM EDTA, and the protease inhibitor cocktail (Complete mini; Roche Diagnostics GmbH, Mannheim, Germany). 10υg of homogenates were electrophoretically separated and transferred to Immun-Blot ^TM^PVDF membrane (Bio-Rad Laboratories, CA, USA) in an electrical field (3mA/cm^2^). Blots were blocked for non-specific protein binding with 5%(w/v) non-fat milk for 1h then incubated overnight at 4°C with anti-MBNL1 rabbit monoclonal antibody (1:1000 dilution, EPR4565, EPITOMICS, CA, USA), and anti-β-ACTIN mouse monoclonal antibody (1:500 dilution, ab8226, Abcam Inc, MA, USA). After repeated washings, the membranes were incubated at room temperature for 30 min with peroxidase labeled anti-rabbit IgG (KPL, MD, USA) or horse radish peroxidase-conjugated anti-mouse IgG (ZYMED, CA, USA) diluted 1:10000. The membranes were then washed and developed to enhance the chemiluminescence with ECL^TM^ Prime Western Blotting Detection Reagent (GE Healthcare, Little Charfont, UK). The chemiluminescence signals were detected with an ImageQuant LAS 4000 mini (GE Healthcare).
