# Supplementary material for: Muscleblind-Like 1 Knockout Mice Reveal Novel Splicing Defects in the Myotonic Dystrophy Brain
Source: PLoS One. 2012 Mar 13;7(3):e33218. doi: 10.1371/journal.pone.0033218 (PMC3302840; doi:10.1371/journal.pone.0033218)
Supplement: Table S1 — Separation score and primers for RT-PCR of mouse brain. (DOC) [file pone.0033218.s006.doc]

| Gene | Gene ID | sep | exon | bp | primer fwd | primer rev |
| --- | --- | --- | --- | --- | --- | --- |
| *Sorbs1* | 20411 | 1.11 | 25 | 168 | ccagctgattacttggagtccacagaag | gttcaccttcataccagttctggtcaatc |
| *Bbs9* | 319845 | 0.99 | 2 | 56 | gctgcttggcaggattctgtctg | ccactgttgtccacatcagccaag |
| *Camk2d* | 108058 | -0.90 | 21 | 89 | catcgcatacattcggctcacac | cgtgtcacatgataagatgacgtgtcac |
| *Camk2d* | 108058 |  | 14-16 |  | cagccaagagtttattgaagaaaccaga | ctttcacgtcttcatcctcaatggtg |
| *Camk2d* | 108058 |  | 14-16 |  | ggaagtccagttcgagtgttcagatga | ctttcacgtcttcatcctcaatggtg |
| *Spag9* | 70834 | 0.63 | 31 | 39 | ggactggaaatggtgtcattatctccat | gggactgccacaaagaatttcacag |
| *Mtdh* | 67154 | -0.62 | 11 | 157 | gacactagagaagagcttccagtgaatacctc | tgtcttccagcactgtgtattctgttgac |
| *Tax1bp1* | 52440 | 0.57 | 10 | 147 | ggcaacacggcaagaacttatctttc | gggtctgtatttattgaagcatcgttca |
| *Acly* | 104112 | 0.51 | 14 | 30 | ccagcacccagtaggacagcatct | cgtctcgggaacacacgtagtcaa |
| *Fcmd* | 246179 | -0.51 | 3 | 138 | cccaagagaacaccatagaccaatgagt | gctatcaaatccaactcgattcccttt |
| *Zmynd11* | 66505 | 0.46 | 4 | 162 | gctggtattgaacaggaaggatattggt | cccatttcctgcttgttagagtgctt |
| *Sorbs1* | 20411 | 0.38 | 6 | 90 | ctgcatctgggaagactcgcct | gacttgctttcatgcttcggagattc |
| *Mprip* | 26936 | -0.37 | 9 | 108 | gcacatggaaaccaacatgctgat | gcttggttagccagcctttcttga |
| *Dclk1* | 13175 | -0.37 | 19 | 74 | gctgtcagtagctggcaaaatcaaga | ctcctcacatcctggttgcgtctt |
| *Mbp* | 17196 | 0.37 | 5 | 78 | cagagacacgggcatccttgact | gggagccataatgggtagttctcgt |
| *Hnrpd* | 11991 | 0.24 | 7 | 147 | ggaacagtatcagcagcagcagca | gcctggatactttcccataaccactct |
| *Nfix* | 18032 | -0.13 | 10 | 148 | ggcaggactcgctgaaggagttt | ctgagactgctgtgggatgttcagaa |
| *Grin1* | 14810 |  | 4 | 63 | tcatcctgctggtcagcgatgac | agagccgtcacattcttggttcctg |
| *Mapt* | 17762 |  | 3,4 | 87,87 | aagaccatgctggagattacactctgc | ggtgtctccgatgcctgcttctt |
| *Mapt* | 17762 |  | 9 | 93 | cccatgccagacctaaagaatgtcag | gcttgtgatggatgttccctaacgag |
| *App* | 11820 |  | 7,8 | 168,57 | caaccaccactgagtccgtggag | gacattctctctcggtgcttggctt |

Mouse Camk2d

Exon 14a: cagccaagagtttattgaagaaaccagatggggtaaag (38 bp)

Exon 14b: aaaaggaagtccagttcgagtgttcagatgatg (33bp)

Exon 15: ataaacaacaaagccaacgtggtaaccagccccaaagaaaatattcctaccccggcgctg (60 bp)

Exon 16: gagccccaaactactgtaatccacaaccctgacggaaacaag (42 bp)

Exon 17: gagtcaactgagagctcaaacaccaccattgaggatgaagacgtgaaag (49 bp)
