# Supplementary material for: Muscleblind-Like 1 Knockout Mice Reveal Novel Splicing Defects in the Myotonic Dystrophy Brain
Source: PLoS One. 2012 Mar 13;7(3):e33218. doi: 10.1371/journal.pone.0033218 (PMC3302840; doi:10.1371/journal.pone.0033218)
Supplement: Table S2 — Primers for RT-PCR of human brain. (DOC) [file pone.0033218.s007.doc]

| Gene | Gene ID | exon | orthologous mouse exon | bp | primer fwd | primer rev |
| --- | --- | --- | --- | --- | --- | --- |
| *SORBS1* | 10580 | 26 | 25 | 168 | ccagctgattacttggaatccacggaag | gttctccttcataccagttctgatcaat |
| *CAMK2D* | 817 | 20 | 21 | 96 | cgccgggatggaaagtggca | gaggagaggacggcccaggg |
| *CAMK2D* | 817 | 14-15^*1^ | 14-16 |  | ttctactgttgcttccatgatg | agtccccattgttgatagctt |
| *SPAG9* | 9043 | 27b^*2^ | 31 | 39 | ggacaggaaatggtgtcattatctccat | gggactgccacaaagaatttcacag |
| *MTDH* | 92140 | 11 | 11 | 157 | gagattagagaagaccttccagtgaatacc | tgtcttccatcactgagtatattctgctg |
| *TAX1BP1* | 8887 | 10 | 10 | 147 | ggcaacacggcaagaacttatctttc | gggtctgtatttattgaagcatcgttca |
| *ACLY* | 47 | 14 | 14 | 30 | ccagcccccagcaggacagcatct | cgtctcgggagcagacatagtcaa |
| *ZMYND11* | 10771 | *3 | 4 | 162 | gctggtattgaacaagaaggatattggt | cccatctcctgtttgtttgtattctt |
| *SORBS1* | 10580 | 5 | 6 | 96 | tggcacctggcagcaatggg | acgaggcccggagagtcacc |
| *MPRIP* | 23164 | 9 | 9 | 114 | gcacatggagaccaatgcagtgg | gcttagtcagccagcctttcttga |
| *DCLK1* | 9201 | 19 | 19 | 74 | gctgtcagtagctggaaagataaaga | ctcctcacatcctggttgcgtctt |
| *MBP* | 4155 | 5 | 5 | 78 | cagagacacgggcatccttgact | gggagccgtagtgagcagttcttgc |
| *MAPT* | 4137 | 3 | 3 | 87 | tacaccatgcaccaagacca | gtctccaatgcctgcttctt |
| *MAPT* | 4137 | 12 | 9 | 93 | actgagaacctgaagcaccag | cacttggaggtcaccttgctc |
| *GRIN1* | 2902 | 4 | 4 | 63 | gtctacagctggaaccacatc | tccatcagcagggccgtcacg |
| *APP* | 351 | 9 | 7 | 168 | ccaccaccacagagtctgtggaa | gacattctctctcggtgcttggcct |

*1: There is a sequence homologous to mouse exon15 in the human gene between human exons 14(a) and 15. The sequence of this exon (14b) is “ataaacaacaaagccaacgtggtaaccagccccaaagaaaatattcctaccccagcgctg”.

*2: There is a sequence homologous to mouse exon 31 in the human gene between exons 27(a) and 28. The sequence of this exon (27b) is “ccgtaatcctccaccagggacgtttactggggctgaggg”.

*3: There is a sequence homologous to mouse exon 4 in the human gene. The sequence of this exon is “gactgggaaacagaaaatcatgactggtattgttttgaatgccatttgcctggagaggtgttgatatgtgacctgtgttttcgtgtgtatcattccaagtgtttgtctgatgagttcaggcttagagacagcagtagtccctggcagtgcccagtttgcagg”.
