# Supplementary material for: Muscleblind-Like 1 Knockout Mice Reveal Novel Splicing Defects in the Myotonic Dystrophy Brain
Source: PLoS One. 2012 Mar 13;7(3):e33218. doi: 10.1371/journal.pone.0033218 (PMC3302840; doi:10.1371/journal.pone.0033218)
Supplement: Table S3 — YGCY motif searching list with sequences exons aberrantly regulated in brains of Mbnl1 knockout mice and DM1 patients. YGCY motifs are indicated by capitals. (DOC) [file pone.0033218.s008.doc]

| Gene |  | exon | 150bp upstream intron +40bp exon | 40bp exon+150bp downstream intron |
| --- | --- | --- | --- | --- |
| *SORBS1* | *human* | 26 | tagttgtggctctacagccgttctgacagctgagtcttaaagcttcTGCCccattcacccagtacctccttccctaattacttttgttggcagttttctaataTGCTggaacagatgattcacTGCCcattttatgcaattttatggcagaaacttttagcggaccagagacgacttaaacgcgagcaag | caatatagacgatactgcaaaaaggaaatctgggtcagaggtctgttttggtcacctcaatcTGCTGCTtgacccaaagcaatattTGCCtattTGCCtcccctcagcattgtcctctgtaggccagtttagttctccaaacagcatctgtcatcctggcctcccttaggctTGCTttgg |
|  | *mouse* | 25 | atagaagttgtgtCCGTaagtccctttgacagtgatctttaatgtctcttccttgttcaccgaTGCCttcctccttagtTGCTtatgtggccattttccgttaagctggaccagatgattcacTGCCtgttttatgcaatttcatggcagaaacttttagcggaccagagacgacttaagcgcgagcaag | caatatagatgatacggccaaaaggaaatctgggttagaggtctgttttggtcacCGCCaacCGCTGCTtgacccaaagcaatattTGCCtttctttggcatTGCCctctgtaggccaatttagttctccaaacagcacctgtcatcctggctttcttagcctTGCTttggctcttttgt |
| *CAMK2D* | *human* | 14b | ttcctgtcagtcatcaccatgtctttcacgttcatcttcagtggctctttatcttcatgtctacatctctatgtagacaTGCTgcatctagagacattggctaccaaggggtttaaggcatctctgtttctattttctgcatacccacagataaacaacaaagccaacgtggtaaccagccccaaagaaa | ggtaaccagccccaaagaaaatattcctaccccagCGCTggtatacctggactctttgtaacccttcttaagttctttacatttttttttttttgtggtttctctcattTGCTttcactatatagatacatatatataaatattttatttcgttgggtgttgggttttgttttttttttttttTGCTttc |
|  |  | 15 | gtcagctgcaactctgacagcagttaacatCGCTttagctcttgtcactatttcattttgtcagctggctcactcTGCTctgtccttcacactagaaagaatgtacttaccaatgcgaactgtttctttctctattttgttctcactcaggagccccaaactactgtaatccacaaccctgatggaaaca | gccccaaactactgtaatccacaaccctgatggaaacaaggtattcagaaagaatcaacatcatctTGCTttaacaagactatacatctcatcaaatattcaaaaaaattaattcgttatctaaaaataggaaagaaaattctaaccattttctaccacacttccttttgggcctaatttatctagaact |
|  | *mouse* | 15 | actagacaTGCTcttcatttgtgtctttctcatccatctccaagggcttttgattctcatatctgtatttctgtataaacacactacttgtagacattggctaccaagaagactaaggcatctctgtttctattttctgcatatccacagataaacaacaaagccaacgtggtaaccagccccaaagaaaa | ggtaaccagccccaaagaaaatattcctaccccggCGCTggtatacctggactcttgcacccttcttaagttctgcactgttTGCTgttgtttctctctttcactaaatcaatatctatgtatcaatacttcatttgttgttggttttgtttgtttgtttgtttgtttTGCTttcaaagatagaagaaaa |
|  |  | 16 | agagatcctgtcagctgttgacagccccttacctggctttagctcttgtcactagtcttgtctgttggccctctcTGCTctgtcccacagactagtaagaatgtacttaccaaaacaaactgtttctttctctattttgttctcactcaggagccccaaactactgtaatccacaaccctgacggaaaca | gccccaaactactgtaatccacaaccctgacggaaacaaggtattcagagagaagcaaccccattTGCTctgataagattacacaactcaccaaaTGCCctgtgcaaaacccagtcattatctcaagtggggaaggaaaaaaaatggccatcttttcaccacaTGCTctttaaggcctaattcttctaga |
| *SPAG9* | *human* | 27b^*2^ | tattaTGCCaagtgggatcttgttattttcgttaggggctttgagcaccaggtttaccagatagaagaCGCTgagagtggtggctcacagtaattctctcctccctgtcatgcacctctccatgtcccctttcctcctttctcactttagccgtaatcctccaccagggacgtttactggggctgagggg | gccgtaatcctccaccagggacgtttactggggctgaggggtaagtgcagattctgaaccaaactcttcttcctggcttctaatagccactgtaaggctgttaccactcctctagaataccctcttgaatgttcctaaatgttatccacttgcagtcactgcatcgcagcaggtgtcttttttttttttt |
|  | *mouse* | 31 | ggccttggtaggaacggtccatcctttggTGCTcTGCCaggctggcatgattcagtagggccatgtagaagaggctcagtgCGCTcacagtaaTGCTtctccttctgtcgtgcacccccccatgtcccctttcctcctttctcactttagccgtaatcctccaccagggacgtttactggggctaagggg | gccgtaatcctccaccagggacgtttactggggctaaggggtaagtgcagatccagaaccaaacttctgtctggcttcttggagccactctgaggctgtctcctcgtctctagagcaccctctccaaTGCTgttcagtgtcacctactcgcagtcactgcattgcagcagaatttgtttttgtttttgtt |
| *SORBS1* | *human* | 5 | ggttatttttaattactgcatctacTGCCctttgtattgttcttgttgtgtgggcgacttcctcctattgtcccTGCCcagttcctcaacTGCCtgagtgtttcccaaatgttctttctggatgcaccatttacccttttgtgttttcagcaacTGCCgaccctttacgcgcaCGCTctatttcTGCTgt | aagacagtgaaaaaCGCCtcaggcctagttctccctacaggtatctccttgggtcggtcgattttctctaactgtgagcggatcatggcatggttttcctttcttcttttcttctaacacgcactttgagctgaatcgttgcatgtttaggaggcattccgtctcacttcctcagctTGCTtccttactt |
|  | *mouse* | 6 | atttttaaggactgcatccacggctctcttcattgttcttgtggcgtaggcagtttcctcctattgtccccacacagttcctcgactTGCCtgagcggctcccaaatgttgtttctggatgcaccgtttaccctcttgtgtttccaacagccgaccctttccgTGCTCGCTctatatcTGCTgttaaaat | aagacagtgaaaagcccttcaggcctagtactccctccaggtatctctctttggggctgtttcttttaattgtccgatcatgcatggtttctttccttcttTGCTcctaacaccatactgtgagccctaatcctcgcatgtgtagaaggcatttcctctccctaagttgtttcctctgtgtgtgcgtgtg |
| *MPRIP* | *human* | 9 | GCTgcagactTGCTatggtggctaggcagatcacTGCCcctCGCCaggagcaaggccctggcggctcttggaggggtggacagcaccagctcgtccctcacaTGCCcacatTGCCctgggctgcactgaccagcggcTGCTctcTGCCacaggacttcaccaatgaagcccccccagctcctctcccaga | caagtcactggacaggaggtccacggagccctccgtgacggtgagcccaggCGCCgcgtcctccggaggccgTGCTcctcTGCTtctggtcactctgaggCGCTgtctgtttctgtctatgcgtccaTGCCagctgtccctctgtgcacaggccctcgtgtccgtgagcacccccacccaccgacccaca |
|  | *mouse* | 9 | gcaTGCCaggacatgggtcatcccagaaccTGCTgtggggcctagacagcacTGCCccactcaaggagggatgaactacccaacttgttgtccccatgcgTGCCcaggcTGCCttgagctgtactgatgggtggcTGCTctcTGCCacaggacttTGCCagcgaagcccccacagctcctctctcagaTG | caagtcattggacaggaggtccacagaatcctccatgacggtgagcccaggccacgTGCCaagttctctggagatctgTGCCactaccacTGCTGCTtctggccactctgggctctgtccatttatgtctttatgtccacactggccctccctctgtaccacccttaTGCCtgtgagTGCCccacccact |
| *DCLK1* | *human* | 19 | atgcagacttaggatattagctagttaccctgcagaagaggcatcgtaactatatatacatatagaatcaatgaagaacagcttggtctggagtcttaattctttagcacaaTGCTgatccactgagtTGCCccatttctctcTGCTcagctggaccacgggtttaccatcaagagatcagggtctttgg | ctttggactactaccagcaaccaggaatgtattggataaggtatgagatggtgcagctcaacagtcagtgtttccatggcaacgTGCTggcagtctccattaaccagttccttcggtggataaacaTGCTtttgatttgttatccagtccataTGCTGCTttatcagtttcattttaatcttgattgaga |
|  | *mouse* | 19 | agattatgttattagctagcTGCTctgcaggaaaggccccatcactgtgtgtgcgtgcgtacgtacgtagttaatgaagagcgtggactggagtcttaattctggaacacaatgttgatccactgagtTGCCccatttctctcTGCTcagctggaccacgggtttaccatcaagagatcagggtctttgg | ctttggactactaccaacaaccaggaatgtattggataaggtatgagatggtgcagctcaacagtcagtgtttccatggcaacgTGCTggcagtctccatttaccagttccttcagtggataaacatgtttttgatttgttacccagtccataTGCTGCTttatcggtttcattttaatcttgattgaga |
